# Supplementary material for: Using association rule mining to jointly detect clinical features and differentially expressed genes related to chronic inflammatory diseases
Source: PLoS One. 2020 Oct 2;15(10):e0240269. doi: 10.1371/journal.pone.0240269 (PMC7531780; doi:10.1371/journal.pone.0240269)
Supplement: Attachment — Submitted filename: ResponseToReviewersPONE-D-20-19597.pdf [file pone.0240269.s005.pdf]

Campinas, August 24th 2020.

RESPONSE TO EDITOR'S AND REVIEWERS' COMMENTS AND SUGGESTIONS

Ref.: "Using association rule mining to jointly detect clinical features and differentially expressed genes related to chronic inflammatory diseases"; by Veroneze et al., submitted to PLOS ONE (PONE-D-20-19597).

The authors would like to thank the reviewers for their helpful and constructive comments and suggestions concerning the aforementioned manuscript. In what follows, we are going to provide the required responses to the raised questions and to describe the initiatives taken to improve the manuscript. Editions made in the manuscript are highlighted in blue.

After the improvements made in this manuscript, we are confident that this study will get the interest of researchers from the many areas involved.

Editor's comments:

1. Please ensure that your manuscript meets PLOS ONE's style requirements, including those for file naming.
The manuscript meets PLOS ONE's style requirements, including those for file naming.

2. We note that you are reporting an analysis of a microarray, next-generation sequencing, or deep sequencing data set. PLOS requires that authors comply with field-specific standards for preparation, recording, and deposition of data in repositories appropriate to their field. Please upload these data to a stable, public repository (such as ArrayExpress, Gene Expression Omnibus (GEO), DNA Data Bank of Japan (DDBJ), NCBI GenBank, NCBI Sequence Read Archive, or EMBL Nucleotide Sequence Database (ENA)). In your revised cover letter, please provide the relevant accession numbers that may be used to access these data.
The data was uploaded to the NCBI repository (https://www.ncbi.nlm.nih.gov/) on August 19 and we are now waiting for an email from GEO curators with our GEO accession numbers, as seen in the attached file 'GEO submission notification.pdf'.

3. We noted in your submission details that a portion of your manuscript may have been presented or published elsewhere:

'The gene expression dataset analyzed in our manuscript was also analyzed in the paper: Corbi et al., Scientific Reports (2020), which was properly cited in our manuscript. In this paper, the authors used traditional bioinformatic tools (Robust Multichip Average, RankProd, Ingenuity Pathway Analysis and Gene Set Enrichment Analysis) to identify differentially expressed genes. Patients' clinical features were not taken into account in the analysis this previous study.'

Please clarify whether this publication was peer-reviewed and formally published.

If this work was previously peer-reviewed and published, in the cover letter please provide the reason that this work does not constitute dual publication and should be included in the current manuscript.

The publication Corbi et al. (2020), Scientific Reports, was peer-reviewed and formally published. Although the patients were the same, the analyses and results were different. The present study submitted to the PLOS One does not constitute dual publication by the following reasons:

**Characteristics**	**The present study submitted to the PLOS ONE**	**Corbi et al., Scientific Reports (2020)**
Data analyses to obtain the Transcriptome (differentially expressed genes, DEG)	---	The raw CEL files were processed using the Robust Multi-array Average (RMA method, as implemented in the Affymetrix package, which were further processed by the RankProd package.
Subsequent analyses developed after the Gene Expression	After obtaining the genes expressed in each patient by the method published by Corbi et al 2020, we utilized Association Rule Mining (ARM) to discover whether there are consistent patterns of clinical features (CFs) together with differentially expressed genes (DEGs) relevant to each group of patients presenting the diseases.	We performed functional enrichment analysis using Ingenuity Pathway Analysis and Gene Set Enrichment Analysis (GSEA). DEGs were submitted to pairwise comparisons, including presentations of networks functionality of genes, and comparisons by Venn diagram of up-regulated genes.
Analysis considering the Clinical Features (CFs) of patients	We utilized ARM to discover whether there are consistent patterns of clinical features (CFs) in each group of patients.	No analysis was performed, only clinical data were reported. All the analyses were centered on the DEGs and functional analyses.
Results	We utilized a different and more powerful method, the ARM method, that can be applied to identify complex multivariate patterns involving clinical and molecular profiles of patients. We obtained 78 CF-rules and 161 CF+DEG-rules. Based on their clinical significance, periodontists and geneticist experts selected 11 CF-rules, and 5	More focused on valided DEGs: Top 20 curated enriched gene sets in circulating lymphocytes and monocytes of each group subject to GSEA. Among validated DEGs verified from T2DMpoorly-DL-P versus H were: TGFB1I1, VNN1, HLADRB4 and CXCL8; T2DMwell-DL-P versus H: FN1, BPTF and PDE3B; DL-P versus

	CF+DEG-rules. We have biologically validated five of the DEGs prospected by the association rules and four of them exhibited a significant difference when compared to the control group.	H: DAB2, CD47 and HLADRB4; P versus H: IGHDL-P, ITGB2 and HLADRB4.

Notice that, in the previous paper of Corbi et al. (2020), we used a distinct set of bioinformatics tools to identify DEGs, and we have not made any association with the clinical features of the patients, thus being a more restricted approach.

In the present study, we utilized a different and more powerful bioinformatics tool, more specifically the process of association rule mining (ARM), a highly interpretable approach which is very effective in identifying complex multivariate patterns when both clinical and molecular profiles of patients are considered. Moreover, the combination of CFs and DEGs can be utilized to better estimate the patient's chance of developing complex diseases, such as those studied here. We biologically validated five of the DEGs prospected by the association rules and four of them exhibited a significant difference when compared to the control group.

Reviewers' comments:

REVIEWER #1: Dear Editor,
I carefully read the manuscript by Veroneze and collaborators, which regards and interesting and timely study.
My comments to improve the paper:
- English language is low-quality and needs to be carefully revised before resubmission.
The manuscript has undergone a careful proofreading.

- Table 1 - Classification of "Dyslipidemia" is substantially wrong and should be reformulated in accordance with the latest international guidelines
We reformulated the analyses according to the 2018 AHA / ACC / AACVPR / AAPA / ABC / ACPM / ADA / AGS / APhA / ASPC / NLA / PCNA Guideline on the Management of Blood Cholesterol, as it can be observed in Table 1 and throughout the text of our manuscript. In the previous version of our manuscript, we considered 3 levels for the total cholesterol (TC) attribute: 1. $TC < 200$, 2. $TC \in [200, 240)$, and 3. $TC \geq 240$. In the current version, we are considering 4 levels: 1. $TC < 150$, 2. $TC \in [150,200)$, 3. $TC \in [200,240)$, and 4. $TC \geq 240$. So, it is now in accordance with the latest international guidelines.

We also included the non-HDL-cholesterol (N-HDL-C) attribute, where N-HDL-C = TC - HDL, in our analysis, since it has been advocated as a good predictor of cardiovascular disease (CVD) risk.

Therefore, we also redid the analysis with the association rule mining (ARM) method and updated the Results and Discussion section of our manuscript.

- References are obsolete and should be updated.
We replaced many references throughout the text, and we also included new and appropriate ones, as recently published as possible. Only two references were not replaced (American Academy of Periodontology [1999] and Löe H. [1993]), because they were used to classify individuals with periodontitis and are classical in the area.

- The flowcharts are unclear.
The flowcharts are now better explained along the text and in the caption of the respective figures.

REVIEWER #2: Dear Authors, there are no doubts in the high actuality of the topic related to exploring an interaction patterns of type 2 diabetes mellitus, dyslipidemia and periodontitis based on its inflammatory background. The use of rule-based machine learning methods for identifying an interaction of clinical and molecular patients profiles adds an additional value. The manuscript is well written, clear and properly referenced.
There are no major issues that could affect the value of the research paper.
If to talk about minor issues, that probably could be taken into consideration for further scientific analysis are:
- relatively small size of the samples and the reasonability to increase the number of patients enrolled in order to get more convincing results for its extrapolation
Regarding the number of patients investigated here, it was necessary three years to appropriately select them because we had many criteria to meet before enrolling each of them. Certainly, increasing the number of patients tends to enhance the strength and impact of the study, and we intend to do that in future studies. Nonetheless, the high confidence of the obtained association rules, based on a data-driven approach, brings additional robustness to the concluding remarks.

- probably, adding in the analysis patients group with type 2 DM and dyslipidemia with TG > 200 mg/ dL and decreased HDL-C (< 38 mg/dL and 46 mg/dL for men and women respectively) could be interesting in order to identify discrepancies with a group of patients with classic diabetic dyslipidemia.
To comply with this, we included an additional analysis comprising only T2DM patients presenting diabetic dyslipidemia, which are patients from Groups 1 and 2 having TG ≥ 204 mg/dL and HDL < 38 mg/dL. The rules found for this pathologic condition are presented in Table 6. We highlight the rule: FPG = 3, HOMA-IR = 2, TC = 2, HDL = 1, TG = 3 → BOP = 3, as it demonstrates that diabetic dyslipidemia is associated with more than 50% tooth site bleeding, one of the main significant signals of periodontium inflammation. Periodontitis is the most common cause of chronic inflammation in diabetic patients. Both periodontitis and diabetes have detrimental effects on each other in terms of alveolar bone destruction and poor metabolic control, by continuous inflammatory mediator activation .

Moreover, not only quantitative, but qualitative changes in lipoproteins could be analyzed as well as adiponectin and leptin levels in order to better characterize clinical profiles.

Indeed, we have interesting results from a previous study enrolling the same patients studied here that showed significantly higher mRNA levels of leptin in dyslipidemic individuals (Groups 1, 2 and 3). Moreover, those leptin mRNA levels were significantly correlated with periodontal parameters such as BOP, suppuration and mainly CAL ≥ 5 mm . We included those findings in the Results and Discussion section of our manuscript.

The authors.
